# A Multi-Component Analysis of CPTED in the Cyberspace Domain

**DOI:** 10.3390/s20143968

**Published:** 2020-07-17

**Authors:** Jemin Justin Lee, Myong-Hyun Go, Yu-Kyung Kim, Minhee Joo, JeongEun Seo, Haengrok Oh, Janghyuk Kauh, Kyungho Lee

**Affiliations:** 1 Center for Information Security Technology (CIST), Korea University, Seoul 02841, Korea; jeminjustinlee@korea.ac.kr; 2 Institue of Cyber Security & Privacy (ICSP), Korea University, Seoul 02841, Korea; mhgo@korea.ac.kr (M.-H.G.); rladb1125@korea.ac.kr (Y.-K.K.); sje5279@korea.ac.kr (J.S.); 3 PUBG Corporation, Korea University, Seoul 06655, Korea; mhjoo9321@pubg.com; 4 Agency for Defense Development (ADD), Seoul 05771, Korea; haengrok@add.re.kr (H.O.); jhkauh@add.re.kr (J.K.)

**Keywords:** lme4, R, access control, natural surveillance, CPTED, FAIR, IoT, spatial syntax

## Abstract

The visual fidelity of a virtual environment lacks the exceedingly complex layers from the physical world, but the continuous improvements of image rendering technology and computation powers have led to greater demands for virtual simulations. Our study employs Crime Prevention through Environmental Design (CPTED) as a risk control measure and utilizes two principles: Access Control and Natural Surveillance. We conducted an experiment with (n-sample: 100) graduate students. For the experiment, we utilized the Factor Analysis of Information Risk (FAIR) to quantitatively analyze the risk. Furthermore, we adopted the lme4 package for R to estimate the mixed effect of the 6,242,880 observations retrieved from Kaggle. Based on the two experiments, we were able to critically evaluate the contributions of CPTED through a multi-component analysis. Our study investigates how spatial syntax and territorial demarcation may translate in the cyberspace realm. We found that the corollaries of the mophology in the virtual environment effects the distribution of crime. The results of our study discusses how to determine the criminogenic designs and capacity in the cyberspace realm.

## 1. Introduction

In the 1930s, Edwin Link sought out to reduce the gap between the quantity and quality of training available for pilots [1]. While the technology for the training simulators was rudimentary, this allowed current pilots in the military aviation to spend more hours on the “flying” simulators than on the real aircraft. Flight simulation have become an integral part of modern combat air operations. Past studies have concluded that stand-alone simulators are incapable of fully meeting the needs of combat medicine or any type of medical training. However, based on the success of the flight simulations, medical commanders have instituted an array of training programs, and the programs have shown varying degrees of success as major initiatives are underway to develop virtual reality products for combat medical training [2]. The U.S. military has launched the National Capital Region Medical Simulation Center under the aegis of the Uniformed Services University of the Health Sciences at the Forest Glen Annex of the Walter Reed Army Medical Center in Maryland [1,3], Army Medical Department Center and School in San Antonio, Texas, Special Operations Medical Academy at Fort Bragg, North Carolina. In recent years, the innovation of game engines has allowed game developers to create game logics and environments that emulate the real world environment. The off-the-shelf products offer an affordable but also immerse experience that can help facilitate the simulation for military and commercial uses [4].

During the past few decades, the surging penetration of various mobile devices, media platforms, and technological advancements have factored into the growth of first-person shooter (FPS) games. With the development and implementation of the fifth generation (5G) LTE network, the 5G LTE network is expected to use a wider range of spectrum allocations than the previous 4G LTE [5]. The number of devices and contents for game consoles, PC’s, and mobile devices is also expected to rise with technologies such as Internet of Things (IoT), Cloud Computing (CC), Virtual Reality (VR) and Augmented Reality (AR). These contents are not limited to streaming contents, online educations, and games. In recent years, FPS games have become a cultural phenomenon as PlayerUnknown’s Battlegrounds (PUBG) sold more than 50 million copies and Fortnite: Battle Royale have generated over $2.4 billion (USD) in the year 2018 [6]. Well established video game franchises such as Call of Duty, Battlefield, and Fortnite have pursued a format that focuses on real-time environments. Consequently, the environment’s interaction with the players have a vital role in the gameplay and overall experience. Moreover, the strong correlation between the advancement of technologies and video game consumption rate has proved that the development of real-time environments will continue to soar. However, the societal dialogue on video games has increased among politicians and news media, as past studies have focused on the relationship between violent video games and violent behavior [7]. Few studies have attempted to reference the mass shootings to video games, even though youth violence has decreased in spite of the recent increase in the video game consumption rates [8].

Even though there have been numerous studies asserting a positive relationship between video games and violence, aggregated crime data proved that claims of video game violence being connected to real-life violence are not supported [8]. Furthermore, space syntax developers have incorporated Oscar Newman’s cul-de-sac design into the urban design. Yet, there have been little to no studies that assess how the defensible space would translate in the cyberspace domain. We posit that introducing environmental criminology from the real-world to the cyberspace realm will exhibit similar patterns. Past studies have employed virtual environments to emulate real-world situations [2]. In order to examine and measure how environmental factors would perform in the cyberspace domain, we tested our studies in the virtual environment.

The visual fidelity of a virtual environment lacks the exceedingly complex layers from the physical world, but the continuous improvements of image rendering technology and computation powers have led to greater demands for virtual simulations [9]. Naturally, these systems’ real-time performance has been improved, and we believe these technological advancements can provide a more immersive environment. Our study aims to examine how the spatial syntax and territorial demarcation may translate in the cyberspace realm. The validation of environmental criminology methods in the FPS gaming context requires a degree of reinterpretation. The simulations within the virtual environment may help policymakers, local authorities, and practitioners to determine the criminogenic designs and capacity in the urban landscape based on the test results.

The remainder of this paper is organized as follows. A Literature review is conducted on the landscape studies of the space syntax studies, Crime Prevention through Environmental Design (CPTED), and Factor Analysis of Information Risk (FAIR). In this study, we posit that the landscape design of the cyberspace domain has a strong correlation with the offensive behavior. Our study is structured as follows. Section 3 iterates upon the data collection method and Section 4 describes our research model and predictions. Section 5 explains the Research Method and Results, and the penultimate is composed on the discussion. Finally, we conclude our paper with the future study and conclusion.

## 2. Research Background

### 2.1. Prior Research on the Notion of Territoriality

The notion of defensible space first introduced how territorial demarcations may elicit and deter prospective criminals from initiating and/or completing acts of crimes [10]. Altman’s typology of human territories has defined the properties based on the dimensions of centrality and temporal duration [11]. According to Altman’s three-fold typology of territorialities, the territories can be divided into: Primary territories, Secondary territories, and Public territories. The hierarchy of defensible space has helped clear the delineation between private and public spaces. The public territories focus on large public areas that often times fail to encourage a sense of ownership. Primary territories refer to the residential areas that are much more personalized, while secondary territories are areas between primary territories and public territories. Oscar Newman’s theory distinguished the territory into private space, semi-public space, and public space [10]. Based on Oscar Newman’s theory and Altman’s typology of territorialities past studies have emphasized how personally revealing defensive markers are much more effective in reducing the number of criminal activities [10,11]. The territorial stratification of the two typologies have provided a clear distinction and demonstrates the degree for the private and public areas.

Prior studies on city design and natural surveillance have found that the environment can reduce crime rates through theoretical and empirical methods [12,13]. Jacobs [12] presented a solution to help solve crimes through the urban redevelopment technique, which is part of a city design method. Jeffory [13] found that studies on crime prevention focuses on models pertaining to deterrence and retribution, however, he contends that we should focus on preventative systems based on scientific principles. Desyllas et al. [14], states that building heights are one of the factors that can affect the crime rates in an urban environment. As such, various elements of the urban landscape will encourage or discourage criminal activities based on the environmental design. Naturally, defining boundaries and maintaining a positive image have been confirmed to help discourage offensive criminal activities. Public safety can be improved through the management of territorial demarcations and environmental design, as territorial demarcations such as the installations closed-circuit television (CCTV) can help function as a security measure for surveillance and a sign that will reduce the criminal’s intentions to act upon their crimes [14]. Crime Prevention through Environmental Design (CPTED) incorporates alienation mechanism within the environment. In the aforementioned Newman’s idea had a major impact on CPTED, since the methodology draws on environmental and behavioral psychology to focus on territorial reinforcement.

### 2.2. Prior Research on Crime Prevention through Environmental Design (CPTED)

Reynald [15] imposes that criminal activities will only take place in the absence of a capable guardian within the context of a residential area. By interviewing a sample of 255 participants, the study proved that capable guardians are able to detect, prevent, or intervene in criminal activities. Studies on environmental criminology have focused on the relationship between environmental factors and criminal activities [16,17,18]. Previous studies on predicting and preventing crimes for residential housing states that providing clarity for the local authorities will allow private developers to avoid implementing criminogenic designs [16]. In the study, 1058 properties were examined by observing the environmental factors associated with the risk of burglary. Agent-based modeling (ABM) is another method that is often used in the urban environment to run simulated scenarios to measure the links between crime events and artificial society. The spatial configuration of London was demonstrated by incorporating the agent-based models [18]. In recent years, CPTED, a multifaceted approach to help reduce crime, has been continuously reviewed and re-examined for crime science [17]. Birks [19] emphasizes that quantifying the crime events is crucial, but the empirical testing of theoretical constructs are currently underdeveloped.

Poyner [20] outlined the four principles of CPTED: surveillance, movement control, activity support, and motivational reinforcement.

1. Surveillance—The first principle of CPTED focuses on increasing the identification and apprehension of criminals or offenders. Providing lighting may help remove possible blind spots, and hiring supervisory personnel such as police officers or security watches. The supervisory personnel can function and help introduce some components of the surveillance.
2. Movement control—Movement control is a measure that intends to limit the movement of the potential offender through the use of barriers such as street closure and placing locks to access the entrance. By restricting the possible boundaries and movements of the offender, the measure may help the offenders refrain from making other movements.
3. Activity support—Increasing the number of attractions and rearranging the facilities to increase human activities within the areas can serve as a measure to help improve the surveillance. By clustering commercial establishments and by adjusting the operating hours, the activity support may increase human activities.
4. Motivational reinforcement—Motivational reinforcement is a tactic that focuses on encouraging personalized environments. By leveraging the involvement of the citizens or community, the public areas are more likely to be better maintained.

**Figure 1:**
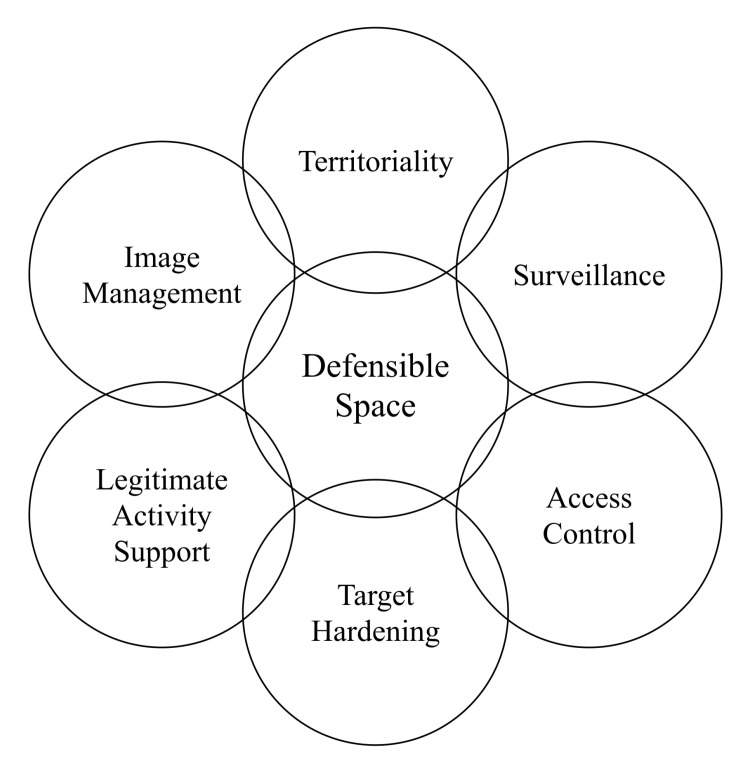
The 1st Generation Crime Prevention through Environmental Design (CPTED) and the seven principles: defensible space, access control, territoriality, surveillance, target hardening, image, and activity support.

The CPTED that is frequently recognized was extended by Cozens [21] by adding seven principles: defensible space, access control, territoriality, surveillance, target hardening, image, and activity support, as depicted in Figure 1.

More recently, Cozens [22] has updated the seven components: territorial reinforcement, natural surveillance, image/space management, natural access control, legitimate activity support, target hardening, and geographical juxtaposition.

Access control is one of the components from CPTED that focuses on denying access to potential targets and creating a heightened perception of the risks in the offenders. Previous studies have proven that there are association between the levels of crimes and design attributes. Access control consists of spatial definition, security personnel, and mechanical strategies. Reduced levels of crimes have been recorded through pedestrian movements on the streets [21]. According to the U.S. Department of Justice, spatial syntax with low levels of lighting, fences, and walls can provide concealment opportunities for the burglars [23].

Surveillance is another component of CPTED that promotes opportunities for guardianship. A certain critical level of population density and movements were linked to criminal activities. Past studies have found that a ‘zone of intensity’ could have a low population density that consists of both victims and offenders, while a higher population density can help mask the burglar’s offenses. A number of researchers have found that offenders refrain from targeting properties with enhanced surveillance and intervisibility [20,21]. Surveillance is a component that can be distinguished into natural/informal surveillance and formal/organized surveillance. The different types of surveillance aim to provide a sense of security to reduce thefts and criminal activities. However, formal surveillance may employ mechanical strategies such as a silent alarm, CCTV, or introduce physical presence through police officers or guards. Yet, natural surveillance does not rely on surveillance that is routinely taking place. Instead, it focuses on surveillance opportunities within the built environment.

### 2.3. Factor Analysis of Information Risk (FAIR)

**Figure 2:**
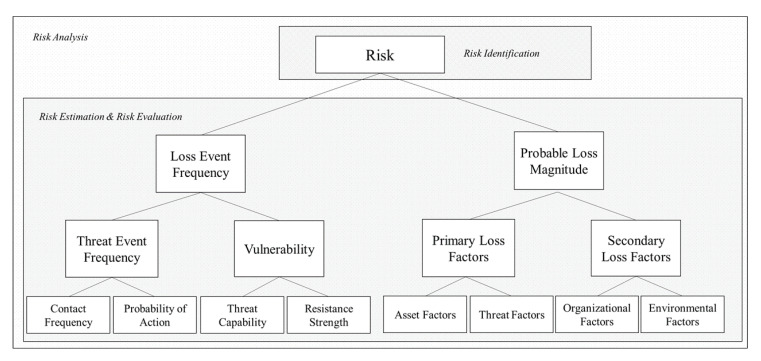
The FAIR (Factor Analysis of Information Risk) Model assesses the organization’s risk by factoring in the Loss Event Frequency (LEF) and Loss Magnitude (LM).

The framework consists of Loss Event Frequency (LEF) and Probable Loss Magnitude (PLM). The framework can be performed using the information gathered using the ISO/IEC 27005 communication framework. The Loss Event Frequency considers the Threat Event Frequency (TEF) and Vulnerability (Vuln). The Probable Loss Magnitude considers the Primary Loss Factors and Secondary Loss Factors. Factor Analysis of Information Risk (FAIR), a taxonomy for information and operational risk, was first conceived by J. A. Jones in the year 2005. FAIR serves as a cyber risk framework that consists of the probable frequency and probable magnitude of future loss. In our study, we account for the Loss Event Frequency (LEF) which consists of the Threat Event Frequency and Vulnerability, as shown in Figure 2. LEF is the probable frequency within a given time frame that loss will materialize from a threat-agent’s action.

A recent study on the feasibility of measuring the risk of IoT devices based on security scenarios concluded that the FAIR is a risk measurement method that is capable of stochastically approaching the measurement of each factor against assets and threats [24]. A study utilizing FAIR proposed a method of assessing Android malware threats through clustering algorithms such as k-Nearest Neighbor (KNN), Support Vector Machine (SVM), and Logistic Regression [25].

## 3. Data Collection

### 3.1. Experiment 1

This paper aims to measure the risk by applying the Risk Management model to the online game environments. Using AssaultCube, an open-source FPS game, we designed two maps for our experiment. The first map, Map1, is the default map provided by AssaultCube. Based on Map1 We changed the exposure rate and altered the environment to create a second map, Map2. To apply the CPTED method to the second map, Map2, we added fences to the environment. By applying contrasting settings to the two different maps, we aimed to observe if the components from Natural Surveillance and Access Control would change the user’s behavior. As mentioned in the aforementioned section, Natural Surveillance has been proven to reduce crime rates. We carried out an experiment with a total of 100 graduate students from the School of Information Security, Korea University, Republic of Korea. We used two laptops with the identical model, LG14Z95, specifications, and settings. The players participating in the game used the same firearm, MF-577. The goal of the experiment was to demonstrate the potential relationship between the natural surveillance and user’s behavior. The participants were given a brief description of the experiment, and a detailed explanation of the control setting was provided prior to the experiment. We conducted a questionnaire survey to analyze the participants response.

The questionnaire survey consisted of questions that pertained to the different maps. We assured the participants that there are no right or wrong answers to ensure that the participants honestly answered the questions. The respondents were asked to recall the situations from the first and second gameplay. We asked the participants to select the words or phrases that best associated with their experiences. The participants were asked to select more than one answer, and they were asked to fill out the answers for both Map1 and Map2. The FAIR model, a risk analysis model, was used in our study to compare the threat and risk between to the two different maps. The model is able to measure the risk by factoring in the probable frequency and probable magnitude of future loss. The FAIR model is dived into LEF and LM. Using the Risk Management Model we proposed a risk management procedure that consists of a five-step process. The first step is ‘determining the scope’ of the risk management. Then the risk analysis is the next step, which consists of ‘identification of the assets’, ‘asset value’, vulnerability evaluation’, ‘threat evaluation’. The ‘risk assessment’ assess and quantifies the risk. The CPTED is finally applied as a control measure.

**Figure 3:**
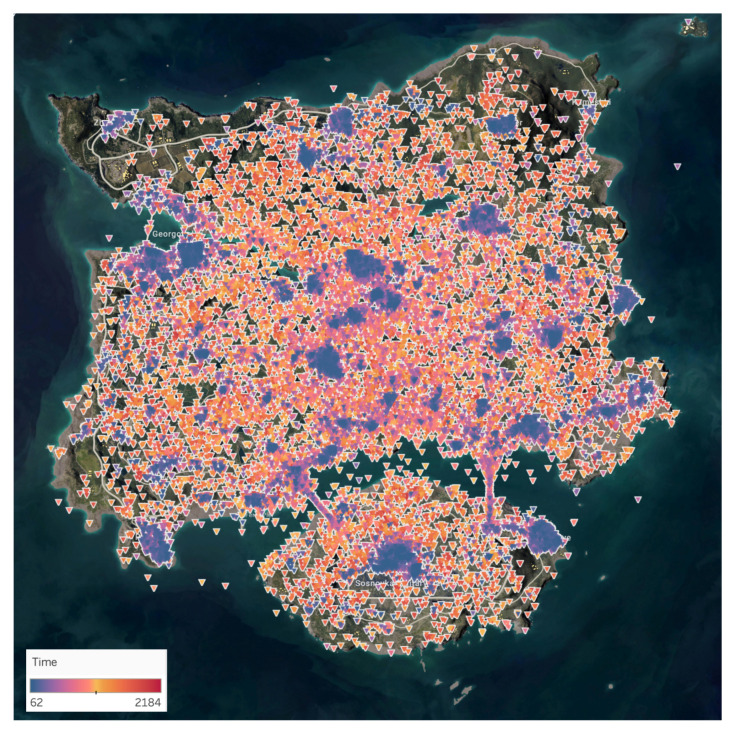
The figure is a visualization of the data clustered by observation for the data placed onto the two-dimensional map.

In our study, we consider the two principles, natural surveillance and access control, from CPTED. Map1 had a low exposure setting in comparison to Map2. By adjusting the brightness within the settings, we were able to apply an aspect of natural surveillance to the Map2, as the brighter environment acted as a natural surveillance to the participants. The fences that were installed within Map2 served as the access control, as shown in Figure 3. As mentioned in the previous section, we conducted a survey after the experiment. The participants consisted of 100 students who received graduate degrees or a college degree. Over 28 percent of the respondents were female, and the 67.1 percent of the participants were in their 20’s and 32.9 percent were in their 30’s. The first question in the survey inquired about the number of bullets the users have used.

### 3.2. Experiment 2

The idiosyncratic nature of PUBG game implies that using PUBG for testing CPTED components requires a degree of reinterpretation and adapting real world assumptions to the first person shooter (FPS) game environment of PUBG. First of all, the element of deterrence operates differently in the FPS context as opposed to the real world. While in the real world deterrence in the personal security context occurs when the subject fears bodily harm, in the FPS context where all players are willingly committing (virtual) acts of violence, deterrence against violence does not exactly abound. Contrary to other FPS games, PUBG is a fitting environment to simulate deterrence, because the rules enforce significant penalties for each delay. So the players, at least in the initial phase of the game, prioritize self-preservation like in the real world. A game session in PUBG has a maximum duration of 40 min—every second counts and this constraint forces the players to act rationally. In addition, PUBG has a dynamic spatial constraint called “blue zone”, which shrinks the game space over time to ensure fast gameplay. Because of this, players first stock up with weapons and supplies, or “loot”, as much and as soon as possible before engaging other players in combat. Players, who can choose the initial location of gameplay, tend to start the game from locations where loot are known to be concentrated.

Naturally, this is a difficult balance to strike for a player since higher concentration of loot will attract more opponents, thereby increasing the chance of prematurely ending the game. Because of this particularity of PUBG, a typical player would avoid sites that increases exposure to opponents while maximizing the chance to collect supplies. But this behavior changes completely after the players have stocked up, as the goal is no longer about self-preservation but to eliminate as many opponents as possible. In sum, players shift from the initial risk averse phase to the risk seeking phase, and then the theory of deterrence no longer holds. While individual time marks for this “phase shift” may vary session to session and by individual preferences, in this study, we limit the initial phase to the first minute (or 60 s) into the game. Our study uses a very conservative approach given that it limits the usable sample size to a significant extent, as it represents only 1/24 of the session’s total duration. Yet, this is a time period in which there is little actualized threats and players prioritize threat avoidance over reward/risk seeking. Henceforth, it is during the first fleeting minute into the game when one can be confident that the players are acting on the perception of risks and threats rather than reacting to them. This is a period in which the components of CPTED in the FPS game setting can be tested rigorously.

#### Data Preprocessing

A set of data extracted from Pubg.op.gg was uploaded on the Kaggle Dataset, which holds data from more than 6 million players from PUBG which is available from “https://www.kaggle.com/skihikingkevin/pubg-match-deaths”. The data consisted of 6,242,880 observations from PUBG’s maps Erangel and Miramar. We focused only on the observations from Erangel, and as such we eliminated the observations from Miramar. We transposed the data obtained from the Kaggle Dataset into a format that we could analyze. Moreover, we removed the outliers using distance-based, density-based method approach. We also eliminated data that had null values within the following metrics: (i) *player id*, (ii) *x, y,coordinate value*, (iii) *maps*. In order to further prepare the data for analysis, each observation is a *revpairwise* relationship between the “killer” and “victim”. Given that a player may eliminate multiple opponents, there are several observations in which the same player (“killer”) is matched with several others (“victim”). In addition, these observations are grouped under game sessions (“match_id”). The number of observations at the two sites are 19,117 and 81,137 for hospital and prison, respectively. After limiting the sample to observations in the first minute (60 s) of the gameplay, we are left with 12,225 observations, which are divided into 2,210 and 10,035 observations at hospital and prison locations, respectively. There is a total of 6623 unique individual players spread across 5271 sessions in the final sample.

## 4. Research Model and Predictions

### 4.1. Access Control

From the Erangel map, we selected two regions, Hospital and Prison. We selected the two regions based on the overall spatial extent, location, and construction. While the two regions share very similar geographical traits, the key difference was the presence of an external fence. Both sites are connected to the main thoroughfare through a single access road of 120 m or so in length. Although entry points to a site in the initial phase are not relevant because players are dropped from the air, exit points matter greatly because they act as chokepoints on the way out of a premise. The presence of a fence/wall around the hospital site with one single exit point is an element of deterrence in the form of access control, in that it raises the chance of a player either being eliminated or wasting too much time while exiting the premise. While this differs from a typical real-world scenario where access control is intended to prevent threats from entering, we believe the element of deterrence remains similar. Because fencing is a form of access control at the hospital, we hypothesize players prefer the prison over the hospital for their favorite initial location. The former has an open layout that allows easier getaway, and players may prefer prison over the hospital to be less risky, both in terms of vulnerability and potential delay in play progress.

We posit that CPTED access control in the PUBG game setting is operating on the players even before the game session starts. Players enter the game via “air drop”, purposefully choosing the starting point of their gameplay. This is a feature different from some other FPS in that initial conditions reflect the players’ assessment of risk. This is yet another reason why one should focus on the first minute of the game, given the fast-paced nature of the play. A player’s choice of the initial location reflects his or her perception of risks and threats, and this ensures that the choice does not take place at random. On the other hand, the operationalization of threat perception in the first minute of the game is complex in that there is an information void in the initial phase of the game. Players are very likely to resort to their previous experience in the game to come up with a subjective assessment of the threat level. This will obviously depend on the players’ experience and skill. The more skilled and experienced, the lower will be the threshold for choosing a site associated with access control, and vice-versa. Essentially, we propose a model in which the effect of access control is demonstrated through location choice. That is, players with lower skill level will avoid choosing a location that presents higher risk of getting eliminated. The present data includes a handy measure that is correlated with the player’s perception of risk, which is the global average of in-game ranking called “placements”. This is measured by looking at the player’s placement in a game session, averaged over all the sessions that he or she participated as included in the Kaggle dataset.

#### Multi-Level Models

We account for the fact that observations are clustered by game sessions or matches by using multilevel regression analysis model [26], while estimating and testing the impact of the player’s threat perception proxied by one’s in-game ranking. If one were to use the standard logistic regression model without accounting for the multiple observations, it may lead to overestimation of the effect and accuracy, given that the variance in the sample data could be underestimated. In order to account for the clustering in the data, we fit a mixed effects model [27] with the intercept and slope as correlated random effects, and the player’s in-game ranking being the sole fixed effect in the model. This is described in Model 3, where the clustering variable is the game session ${\mathrm{match}}_{id}$.

(1) $$\frac{\log\pi_{ij}}{\left(1-\pi_{ij}\right)}=\beta_{0j}+\beta_{1}x_{1}.$$

Model 1 can be expressed as Equation (1). The latent group characteristics can be explained by group variables. We chose the cluster variable to be the game session, because it implicitly encompasses both *killers* and *victims* whereas individual player clustering is a lesser grouping criterion that does not make that distinction. $\frac{\log\pi_{ij}}{\left(1-\pi_{ij}\right)}$ = $\beta_{0j}+\beta_{1}x_{1}$ where $\pi_{i}$ = P(Y(location)) =$1\left(hospital\right)|x_{1})$ and $x_{1}$ is the player *i*’s is the average global ranking in the kaggle dataset from Equation (1).

(2) $$\frac{\log\pi_{ij}}{\left(1-\pi_{ij}\right)}=\beta_{0j}+\beta_{1}x_{1}+R_{ij},$$

(3) $$\beta_{0j}=\gamma_{00}+U_{0j},$$

(4) $$U_{0j}\;\sim N\left(0,\tau_{00}^{2}\right)R_{ij}\;\sim N\left(0,\sigma^{2}\right).$$

From Model 1, we can derive to Model 2, which can be expressed as Equations (2) and (3). The observations and game sessions can be expressed as, *i* (observations) = 1, … *i* …, 12,225 and *j* (game sessions) = 1, … *j* …, 5,271. The coefficients in the one-level model can become dependent variables in two-level regression models for Model 3, as expressed in Equations (5) and (6). The *U* terms $U_{0j}$ and $U_{1j}$ in Equations (3) and (6) are residual terms at the class level. Equation (5) is a one-level regression model, and Equation (6) uses a two-level regression model with random intercepts and slopes. The random intercepts and slopes effects for Model-3 can be expressed as Equation (7).

(5) $$\frac{\log\pi_{ij}}{\left(1-\pi_{ij}\right)}=\beta_{0j}+\beta_{1}x_{1j}+R_{ij},$$

(6) $${}_{\beta_{1j}=\gamma_{10}+U_{1j}}^{\beta_{0j}=\gamma_{00}+U_{0j}},$$

(7) $$\left(\begin{array}{c} U_{0j}\\ U_{1j} \end{array}\right)∼N\left(\begin{array}{cc} 0 & \tau_{00}^{2}\tau_{01}\\ 0 & \tau_{01}\tau_{10}^{2} \end{array}\right);R_{ij}∼N\left(0,\sigma^{2}\right).$$

### 4.2. Natural Surveillance

**Figure 4:**
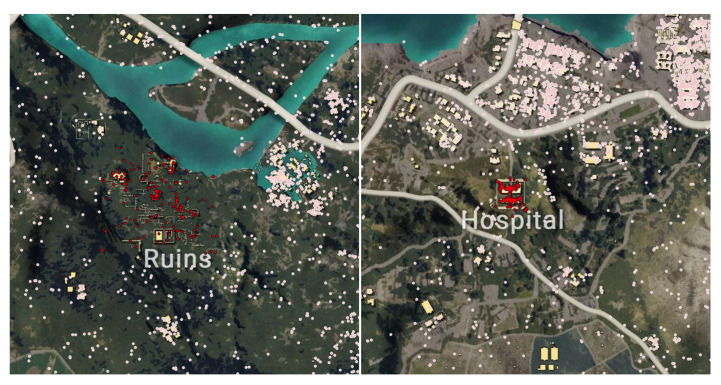
The locations ‘Ruins’ and ‘Hospital’ demonstrate how CPTED can be applied using a single component, natural surveillance. The Ruins offers players a more disperse and open area, while the hospital provides a fence and building. The two locations have similar death counts. Ruins consists of 156 observations, and the Hospital has a total of 167 observations. While the two areas share similar observations of the victim’s death, the two locations offer different forms of clusters.

Natural surveillance is one of the seven principles of CPTED that allows offenders to perceive the potential for intervention, apprehension, and prosecution. Past studies have proved that low levels of lighting and high walls can provide concealments for the offenders [21,22,28]. As such, we posit that areas such as the open road and plane field are more susceptible for surveillance. Our study cross examined regions that have elements of the natural surveillance to those that does not contain the element, as shown in Figure 4. Using distance mapping is substantially based on the ideal Euclidean metric. For each iterations the labels can only propagate at a distance assuming the physically parallel logic is employed for each logic. Based on the Euclidean distance, we were able to able to find the distance between the victim’s *x*, *y* coordinate values, $\left(v_{1},v_{2}\right)$, and killer’s *x*, *y* coordinate value $\left(k_{1},k_{2}\right)$, as expressed in Equation (8).

(8) $$d_{s}\left(i,j\right)=\sqrt{{\left(v_{1}-k_{1}\right)}^{2}+{\left(v_{2}-k_{2}\right)}^{2}}$$

## 5. Research Method and Results

### 5.1. Results from Experiment 1

In our study, we consider the two principles, natural surveillance and access control, from CPTED. We utilized two maps with different settings. The first map, Map1, had a low exposure setting in comparison to the second map, Map2. By adjusting the brightness within the settings, we were able to apply natural surveillance to the Map2. The brighter environment served as a component of natural surveillance to the participants. We also introduced fences in the second map that served as an access control component. Map1 is a default map that is available within the game, but we introduced a few components from CPTED to Map2. The experiment was conducted with the intention to measure the risk by applying the Threat Event Frequency (TEF) and Vulnerability (Vul) from FAIR framework. In the experiment, we classified the risks into five different levels: (i) Very High, (ii) High, (iii) Moderate, (iv) Low, and (v) Very Low. We distinguished the five different levels based upon the number of bullets fired. When the number of bullets that were shot was greater or equal to 30, the participant’s TEF was assigned a ‘Very High’ level. A ‘High’ level was assigned to the user when they shot less than 30 rounds of bullets, but greater or equal to 20. The ‘Moderate’ grade was assigned when the user fired less than 20 bullets, but fired greater or equal to 10 bullets. The ‘Low’ level was assigned to the users who shot less than 10 bullets, but shot greater or equal to 5 bullets. The ‘Very Low’ grade was assigned to the users who had used less than 5 bullets.

**Figure 5:**
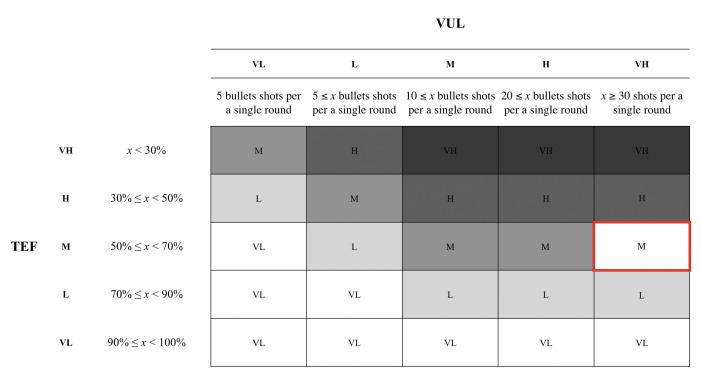
The heat map for Map1 was assigned a ‘Moderate’ grade based on the participants’ response on the TEF and Vul.

**Figure 6:**
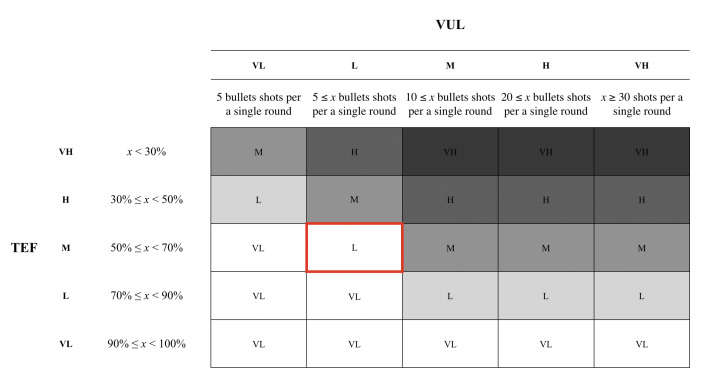
The heat map for Map2 scored a ‘Low’ grade based on the participants’ response on the Threat Event Frequency (TEF) and Vulnerability (Vul).

For this study, we categorized a list of words that are associated positive and negative emotions. Each word was assigned a probability value associated with either positive or negative values. Based on the words that the participant selected, we were able to calculate the value of the vulnerability (Vul). In order to calculate the vulnerability, we assigned five different grade to the value. Based on the words that the participant selected, we were able to calculate the value of the vulnerability (Vul). In order to calculate the vulnerability, we assigned five different grade to the value. Based on the results from both the threat event frequency and vulnerability, we were able to calculate the risk. The participants assigned Map1 a ‘Very High’, however, Map2 had a very opposite grade by scoring a ‘Low’. Based on the results, we were able to ascertain that applying the CPTED method to the map provides a more positive ambiance to the overall gaming experience, as shown in Table 1. Based on the result of the TEF and Vul, we calculated the risks using a heat map for both the first and second maps. The participants have assigned a ‘Moderate’ grade for the risk associated with Map1 as shown in Figure 5. Yet, Map2 scored a ‘Low’ as the environment reflected a more positive feeling towards the environment, as depicted in Figure 6. The participants were asked to fill out a questionnaire that inquires about the user’s demographic information and experience with Map1 and Map2.

### 5.2. Results from Experiment 2

**Figure 7:**
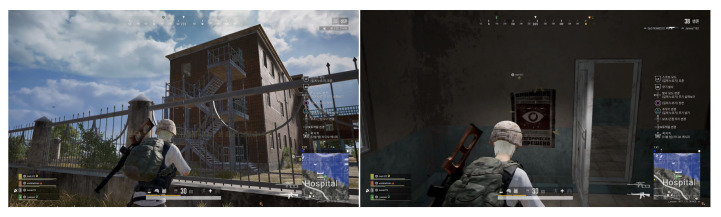
The figure on the left is a screenshot of PlayerUnknown’s Battlegrounds (PUBG) gameplay within the Hospital’s vicinity. The figure on the right is a screenshot of PUBG gameplay within the building in the Hospital area.

**Figure 8:**
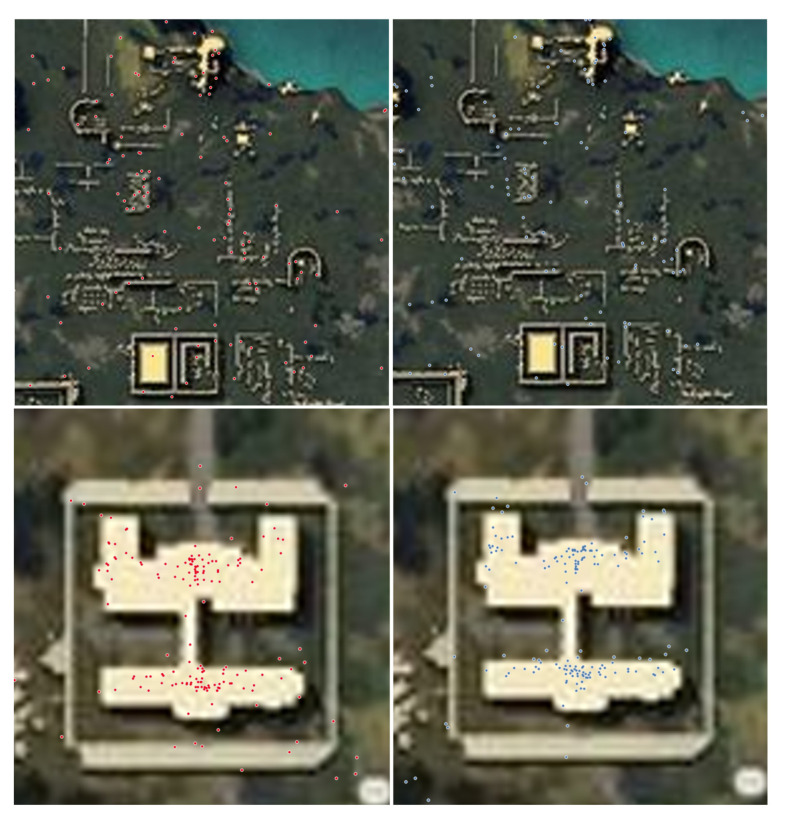
(On the upper right and upper left figure, the figure illustrates the victims (red) and killers (blue) within the ‘Ruin’ area. On the lower right and upper left figure, the figure illustrates the victims (red) and killers (blue) within the ‘Hospital’ area.

Natural surveillance is one of the seven principles of CPTED that allows offenders to perceive the potential for intervention, apprehension, and prosecution. Our study proved that low levels of lighting and high walls can provide concealments for the offenders. Based on the results from the distance mapping, we found that open roads and plane field are more susceptible for surveillance for the users. The cluster patterns from the different regions clarifies how the open area between the building and fence creates intervisibility and physical deterrence for the users. The building structure from the hospital provides concealment for the players, while the open area outside the hospital building creates an environment that can adopt natural surveillance, as shown in Figure 7. Since the environment in the Ruins is an open landscape that offers very limited concealment for the players, the victim areas within the Ruins depicts a more distributed cluster. Contrary to the open landscape of the Ruins, the Hospital illustrates a more condensed cluster around the hospital building. The fences within the Hospital does not mask the players from the other opponents. As such, the players are seen within the building to increase their chance of survival. Our study proved that open areas create an enhanced surveillance opportunity, as illustrated in Figure 8.

The result shows that the association between a player’s location choice and the player’s threat perception is statistically significant with a p-value less than 0.01 (see Model 3 results below). A more simplistic general linear model (GLM) without random effects estimates that for one unit decrease in gaming ranking (because higher is worse), the chance of choosing hospital as location goes down by approximately 6%. But the final model (Model 3) shows that is an overestimation, possibly reflecting the fact that in some game sessions players’ skills are highly unequal, whereas in others they are similar. Accounting for this, the probability of choosing hospital is halved to 3% per unit decrease in ranking. The comparison between Table 2, Table 3, Table 4 and Table 5, which only has the intercept as the random effect for the game sessions, shows that the threat perception (as proxied by average game ranking) is dependent upon the characteristics of individual game sessions. This is meant to be: while threat perception is operationalized in this context by the player’s own ranking, PUBG player matching algorithm tends to compose game sessions with players of similar skill levels. So some sessions would tend to be composed of players with high skill whereas for others the opposite would be true. Model 3, as specified, takes into account this heterogeneity. Nonetheless, the result shows that threat perception affects location choice in a significant manner and as predicted. Given that this association between access control and threat perception has been validated over 5000 game sessions, it gives a high degree of confidence that such an association is not a fluke but rather a validation of CPTED in the FPS gaming context.

## 6. Discussion

The idiosyncratic nature of PUBG game implies that using PUBG for testing CPTED components requires a degree of reinterpretation and adapting real world assumptions to the first person shooter (FPS) game environment. The element of deterrence operates in a different manner in the FPS context, as opposed to the real world context. While in the real world deterrence in the personal security context occurs when the subject fears bodily harm, in the FPS context where all players are willingly committing acts of violence, deterrence against violence does not exactly abound. Contrary to other FPS games, PUBG is a fitting environment to simulate deterrence, because the rules enforces significant penalties for each delay.

The two regions in PUBG, Hospital and Prison, are useful comparisons because they are broadly similar in their overall spatial extent, location, and construction, but with the key difference being the presence of an external fence. Both sites are connected to the main thoroughfare through a single access road of 120 meters or so in length. Although entry points to a site in the initial phase are not relevant because players are dropped from the air, exit points matter greatly because they act as chokepoints on the way out of a premise. The presence of a fence/wall around the hospital site with one single exit point is an element of deterrence in the form of access control, in that it raises the chance of a player either being eliminated or wasting too much time while exiting the premise. While this differs from a typical real-world scenario where access control is intended to prevent threats from entering, we believe the element of deterrence remains similar. Since fencing is a form of access control at the hospital, we hypothesized players prefer the prison over hospital for their favorite initial location. The former has an open layout that allows easier getaway, and players may prefer prison over hospital to be less risky, both in terms of vulnerability and potential delay in play progress. We posit that CPTED access control in the PUBG game setting is operating on the players even before the game session starts. Players enter the game via “air drop”, purposefully choosing the starting point of their gameplay. This is a feature different from some other FPS in that initial conditions reflect the players’ assessment of risk. This is yet another reason why one should focus on the first minute of the game, given the fast-paced nature of the play. A player’s choice of the initial location reflects his or her perception of risks and threats, and this ensures that the choice does not take place at random.

On the other hand, the operationalization of threat perception in the first minute of the game is complex in that there is an information void in the initial phase of the game. Players are very likely to resort to their previous experience in the game to come up with a subjective assessment of the threat level. This will obviously depend on the players’ experience and skill. The more skilled and experienced, the lower will be the threshold for choosing a site associated with access control, and vice-versa. Essentially, we propose a model in which the effect of access control is demonstrated through location choice. That is, players with lower skill level will avoid choosing a location that presents a higher risk of getting eliminated. The present data includes a handy measure that is correlated with the player’s perception of risk, which is the global average of in-game ranking called “placements”. This is measured by looking at the player’s placement in a game session, averaged over all the sessions the players participated as included in the Kaggle dataset.

## 7. Conclusions

In the past decade, the gaming industries have been able to create virtual environments that can replicate environments that are similar to the real world. The technological advancements have influenced the user’s gameplay and environment, as FPS games have incorporated new formats and technologies to the game environment. However, academic literature on the security assessment for the environmental design in the virtual space is limited, as most of the studies have focused on deterring criminal offenses or reviewing the effectiveness of various security measures. Our study utilized the FAIR model to assess the risk and examines the environment within the virtual space by applying CPTED. Our work explores how components from CPTED, such as Natural Surveillance and Access control, can have an effect on the user’s behavior within the spatial syntax. The advancement and innovation have allowed various online games such as First-Person Shooter (FPS) games to garner much attention. As such, we were able to examine how the components of CPTED could influence the cluster distribution and perceived risks of the users.

Our study employs CPTED as a risk control measure and utilizes two principles: Access Control and Natural Surveillance. We conducted an experiment with (n-sample: 100) graduate students. We adopted the Factor Analysis of Information Risk (FAIR) to quantitatively analyze the risk. Furthermore, we adopted the lme4 package for R to estimate the mixed effect of the 6,242,880 observations retrieved from Kaggle. Based on the two experiments, we were able to critically evaluate the contributions of CPTED through a multi-component analysis. Our study focuses on analyzing CPTED within the Cyberspace realm. To the best of our knowledge, our paper is one of the first studies to apply CPTED to a virtual environment. We believe our study was able to provide a better understanding and insight towards the FAIR model and CPTED. This underscores the need to employ techniques and approaches inkling ML techniques and approaches. We also intend in our future work to study the use of our proposed approach through machine learning algorithms.

The visual fidelity of the cyberspace environment lacks the exceedingly complex layers from the physical world, but the continuous improvements of image rendering technology and computation powers have led to greater demands for virtual simulations. Conversely, the real-time performance of these systems have been improved, and we believe these technical advancements can provide a more immersive environment. We believe our study can help future researchers, landscape designers, criminologist, and neuroscientists to gain a better understand of how spatial syntax and territorial demarcation may translate in the cyberspace realm. The simulations within the virtual environment may help policymakers, local authorities, and practitioners to determine the criminogenic designs and capacity in the urban landscape based on the test results.

## Figures and Tables

**Table 1:** The *Vulnerability (Vul)* value for both *Map1* and *Map2*.

Rating	Map1	Map2	Description
Very High(VH)	X		x≥30 bullets per one game
High(H)			20≤x bullets per one game
Moderate(M)			10≤x bullets per one game
Low(L)		X	5≤x bullets per one game
Very Low(VL)			5 bullets per one game

**Table 2:** Model 1: glm(formula = yloc $\tilde{k}$ p$mean_rank, family = binomial(link = “logit”)).

Coefficients:	Binomial (Logit)				
	Estimate	Std. Error	z Value	Pr(>|z|)	
(Intercept)	−1.358742	0.039491	−34.406	<2e-16	***
kp$mean_rank	−0.006108	0.001291	−4.731	2.24e-06	***
Signif. codes:	0 ‘***’	0.001 ‘**’	0.01 ‘*’	0.05 ‘.’	0.1 ‘ ’ 1
Null deviance:	11,562	on 12,244	degrees of freedom		
Residual deviance:	11,539	on 12,243	degrees of freedom		
AIC:	11,543				
Number of Fisher Scoring iterations:	4				

**Table 3:** Model 2-0: Generalized linear mixed model fit by maximum likelihood (Laplace Approximation) [’glmerMod’].

Family:	Binomial				
Formula:	yloc 1 + (1|kp $match_id)				
	AIC	BIC	logLik	deviance	df.resid
	3352.1	3366.4	−1674.1	3348.1	9134
Random effects:	Groups	Name	Variance	Std. Dev.	
	kp $match_id	(Intercept)	1319	36.31	
Number of obs:	9136		Groups:	kp $match_id, 4264	
Fixed effects:	Estimate	Std. Error	z value	Pr(>|z|)	
(Intercept)	−11.8306	0.2848	−41.53	<2e-16 ***	

**Table 4:** Model 2: Generalized linear mixed model fit by maximum likelihood (Laplace Approximation) [’glmerMod’].

Family:	Binomial (Logit)				
Formula:	yloc kp $mean_rank + (1|kp $match_id)				
	AIC	BIC	logLik	deviance	df.resid
	4804.9	4827.2	−2399.5	4798.9	12,242
Random effects:	Groups	Name	Variance	Std. Dev.	
	kp $match_id	(Intercept)	1108	33.29	
Number of obs:	12,245		Groups:	kp $match_id, 5271	
Fixed effects:	Estimate	Std. Error	z value	Pr(>|z|)	
(Intercept)	−11.399458	0.267476	−42.619	<2e-16 ***	
kp $mean_rank	−0.006802	0.005433	−1.252	0.211	
Signif. codes:	0 ‘***’	0.001 ‘**’	0.01 ‘*’	0.05 ‘.’	0.1 ‘ ’ 1
Correlation of Fixed Effects:					
kp$mean_rank −0.485					

**Table 5:** Model 3: Generalized linear mixed model fit by maximum.

Family:	Binomial (Logit)				
Formula:	yloc kp$mean_rank $mean_rank + (kp$mean rank | kp $match_id)				
	AIC	BIC	logLik	deviance	df.resid
	4679.4	4716.5	−2334.7	4669.4	12,240
Scaled residuals:	Min	1Q	Median	3Q	Max
	−2.3833	−0.0030	−0.0024	−0.0015	3.5643
Random effects:	Groups	Name	Variance	Std. Dev.	
	kp $match_id	(Intercept)	1108	33.29	
Number of obs:	12,245		Groups:	kp $match_id, 5271	
Fixed effects:	Estimate	Std. Error	z value	Pr(>|z|)	
(Intercept)	−11.08337	0.36542	−30.331	< 2e-06 ***	
kp $mean_rank	−0.03604	0.01198	−3.008	0.00263 **	
Signif. codes:	0 ‘***’	0.001 ‘**’	0.01 ‘*’	0.05 ‘.’	0.1 ‘ ’ 1
Correlation of Fixed Effects:					
kp$mean_rank −0.739					

## Abbreviations

The following abbreviations are used in this manuscript:

CPTED	Crime Prevention Through Environmental Design
FAIR	Factor Analysis of Information Risk
IoT	Internet of things
FPS	First-Person Shooter
PUBG	PlayerUnknown’s Battlegrounds
GLM	General Linear Model
TEF	Threat Event Frequency
Vul	Vulnerability
LEF	Loss Event Frequency
PLM	Probable Loss Magnitude
